# The Transactions for 1890

**Published:** 1890-10

**Authors:** 


### TRANSACTIONS FOR 1890.

The Transactions of the Texas State Medical Association for 1890 has been published in a handsome octavo volume and bound in neat purple cloth and gold, uniform with the preceding volumes. Six hundred and fifty copies were printed. There are in round numbers five hundred members of the Associatlon, and it takes about one hundred and fifty to supply the medical press, State medical societies, public libraries, etc. It is a handsome volume, and the contents will compare well with any preceding issue.

Copies have been sent to all active members who have paid their dues, to honorary members, and to the leading medical journals, secular press, sister associations, libraries, etc., to the number of about four hundred and fifty.

### PARTICULAR NOTICE.

The Secretary requests the JOURNAL to announce that there are one hundred and fifty-six copies in his office, wrapped, tied and addressed to members who have so far failed to pay their dues for the current year; and that by resolution he is prohibited from mailing them until all arrears are paid. Upon receipt of the amount due from any of these members, or upon notice from the Treasurer that any one has remitted, the Secretary will be pleased to put 14 cents on his volume and mail it. Don't blame the Secretary; he would be unworthy of your confidence did he not carry out the instructions of the Association.

Diuretine, New Diuretic.—This agent purports to be a species of double salt—a salicilate of theobromine and sodium. It is soluble in half its weight of water. The adult dose recommended is about 15 grains and may be repeated five or six times in the twenty-four hours.—London Correspondent *Journal American Medical Association*, August 26, 1890.

